# A new method for achieving enhanced dielectric response over a wide temperature range

**DOI:** 10.1038/srep15144

**Published:** 2015-10-19

**Authors:** Deepam Maurya, Fu-Chang Sun, S. Pamir Alpay, Shashank Priya

**Affiliations:** 1 Bio-inspired Materials and Devices Laboratory (BMDL), Center for Energy Harvesting Materials and Systems (CEHMS), Virginia Tech, 24061 USA; 2 Department of Materials Science & Engineering, Department of Physics, Institute of Materials Science, University of Connecticut, Storrs, CT 06269-3136, USA

## Abstract

We report a novel approach for achieving high dielectric response over a wide temperature range. In this approach, multilayer ceramic heterostructures with constituent compositions having strategically tuned Curie points (*T*_C_) were designed and integrated with varying electrical connectivity. Interestingly, these multilayer structures exhibited different dielectric behavior in series and parallel configuration due to variations in electrical boundary conditions resulting in the differences in the strength of the electrostatic coupling. The results are explained using nonlinear thermodynamic model taking into account electrostatic interlayer interaction. We believe that present work will have huge significance in design of high performance ceramic capacitors.

High dielectric constant and temperature stability are extremely important for ceramic capacitor industry. Due to the development of new high performance electronic materials, the working temperature range of electronic components and devices has been consistently increasing. The high performance capacitor required for ever shrinking electronics should have high dielectric constant with high thermal stability. There have been many efforts to improve the dielectric constant of capacitor materials and their temperature stability primarily using dopant engineering^1^,^2^,^3^. This approach utilizes the high dielectric constant at the Curie temperature, which can be further manipulated by compositional modifications and shifted towards room temperature. However, this in many cases induces increased temperature dependent behavior of dielectric response measured in terms of temperature coefficient. Further, in an approach to achieve flattening of the Curie peak for X7R capacitors, synthesis of core-shell grain microstructure has been performed^4^,^5^. In the core-shell microstructure, depending upon the connectivity, the dielectric properties were considered to be dominated by the continuous or the semi-continuous shell volume having micro-volumes of different compositions, and therefore spread of Curie temperatures represented by Gaussian distribution^4^. The dielectric response of the core-shell structure has been modeled using semi empirical logarithmic mixing law^4^. Despite these prior efforts, achieving high dielectric constant with enhanced temperature stability still remains challenging^6^,^7^,^8^,^9^,^10^,^11^,^12^. Here, we demonstrate high temperature stability of dielectric response in multilayer composite architecture that consists of several different compositions with varying Curie temperatures and a suitable electrical boundary condition. In combination with the graded architecture this composite structure imparts the high dielectric constant in a wide temperature range with excellent stability. We modelled this behavior to reveal fundamental laws governing the interaction among the heterolayers. The method proposed here will be very effective in achieving high dielectric constant in a wide temperature range for advanced capacitor applications requiring precise operation and high temperature stability. The high dielectric constant will further help in miniaturization of devices leading to high volume efficiency.

Various lead-free compositions have been studied to provide high dielectric constant materials including, BaZrO_3_^6^, 0.7BaTiO_3_-0.3BiScO_3_^7^, BiScO_3_-BaTiO_3_-Bi_0.5_K_0.5_TiO_3_^8^, 0.9(B_0.5_Na_0.5_)TiO_3_-0.1KTaO_3_^9^, and other compositions^10^,^11^,^12^. Most of these materials were found to demonstrate low permittivity, high losses, and strong temperature and frequency dependent properties. Moreover, a major fraction of new lead-free capacitor materials encompass alkali-based lead-free ceramic materials (traditionally BaTiO_3_ has been the backbone of ceramic capacitors). Due to volatile nature, the presence of alkali elements makes it quite challenging to process them at high temperature, and hence, hinders their industrial exploitation. Additionally, cost of niobium in alkali based compositions also poses some constraints. Here, we first developed novel compositions with strategically tuned Curie temperatures. Next, we integrated these materials into composite architecture with well-defined electrical boundary conditions to achieve high dielectric constant and wide temperature stability.

## Results

**Figure 1:**
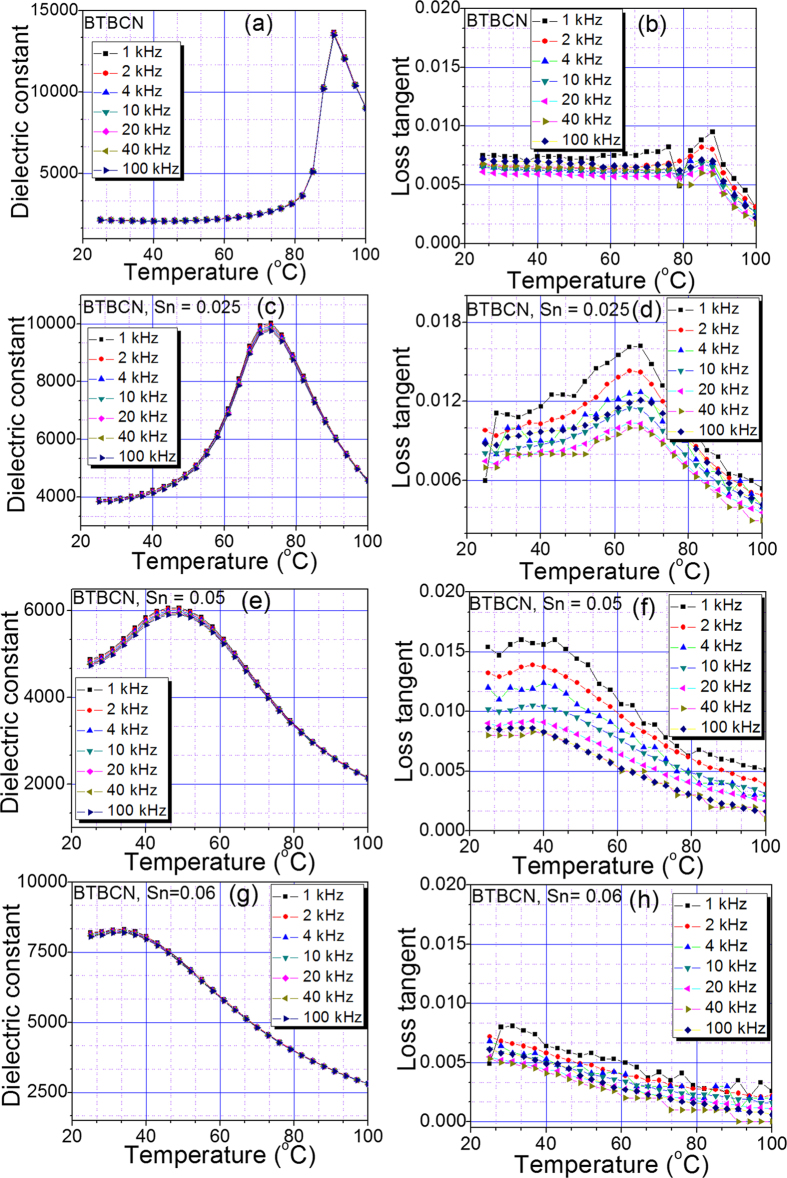
The temperature dependence of the dielectric response for various constituent compositions of laminate composites. Temperature dependence of dielectric constant and loss tangent at various frequencies for BTBCN with: (**a**,**b**) Sn = 0.0, (**c**,**d**) Sn = 0.025, (**e**,**f**) Sn = 0.05, (**g**,**h**) Sn = 0.06.

**Figure 2:**
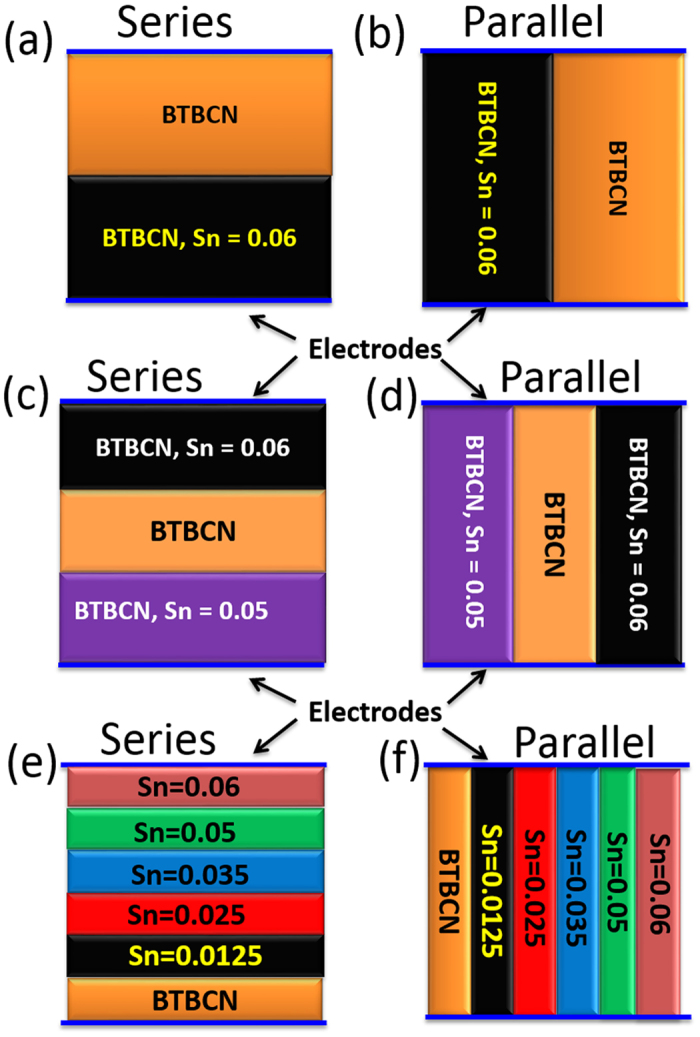
Schematic representations of various ceramic composites fabricated in this work. Series and parallel structures of (**a**,**b**) bilayer, (**c**,**d**) trilayer, (**e**,**f**) hexa-layer composites. The schematic representation are not to scale.

Figure 1 shows the dielectric constant as a function of temperature at various frequencies for pure and Sn-doped derivatives of BaTiO_3_-Ba(Cu_1/3_Nb_2/3_)O_3_ (BTBCN). From this plot, one can clearly observe the shifting of Curie temperature towards room temperature. This shift in Curie temperature was accompanied by the increase in the room temperature dielectric constant. Moreover, the Curie peak was rather broad for heavily doped compositions. This is expected considering the fact that substitution of Ti^+4^ site by Cu^+2^, Nb^+5^, and Sn^+2^ will result in heterogeneity creating point defects as required for charge compensation. Interestingly all the compositions show very small value of loss tangent factor (<1.6%) over a wide temperature range including phase transitions, which is one of the important parameter for high performance capacitor. These results suggest BTBCN and its doped derivatives are quite suitable for ceramic capacitor applications. Next, we utilized these compositions to synthesize multilayer structures for ceramic composite capacitors. We fabricated bilayer, trilayer and multilayer with varying compositions and performed dielectric measurements in parallel and series electrical configurations as shown in schematic representation in Fig. 2.

**Figure 3:**
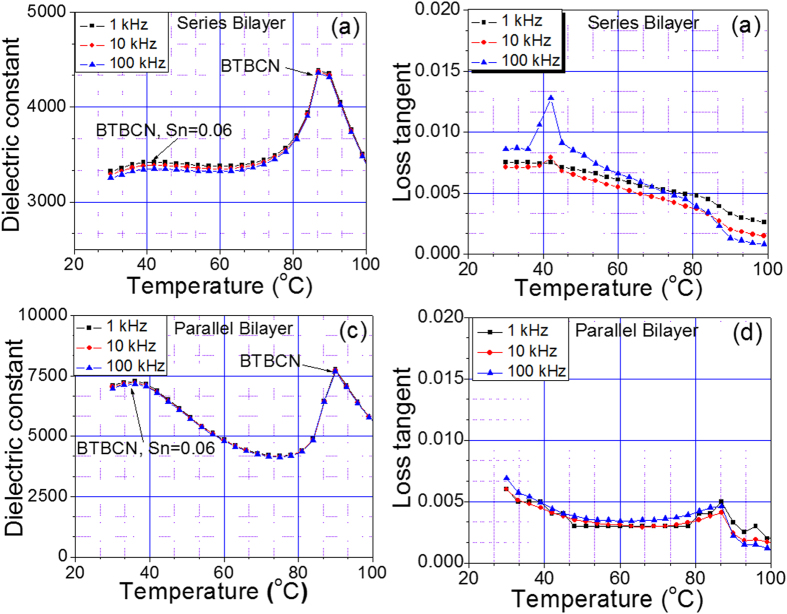
The phase transition behaviour for bilayer laminate composites. The dielectric constant and loss tangent versus temperature plots at various frequencies for bilayer composites in: (**a**,**b**) series configuration, (**c**,**d**) parallel configuration.

**Figure 4:**
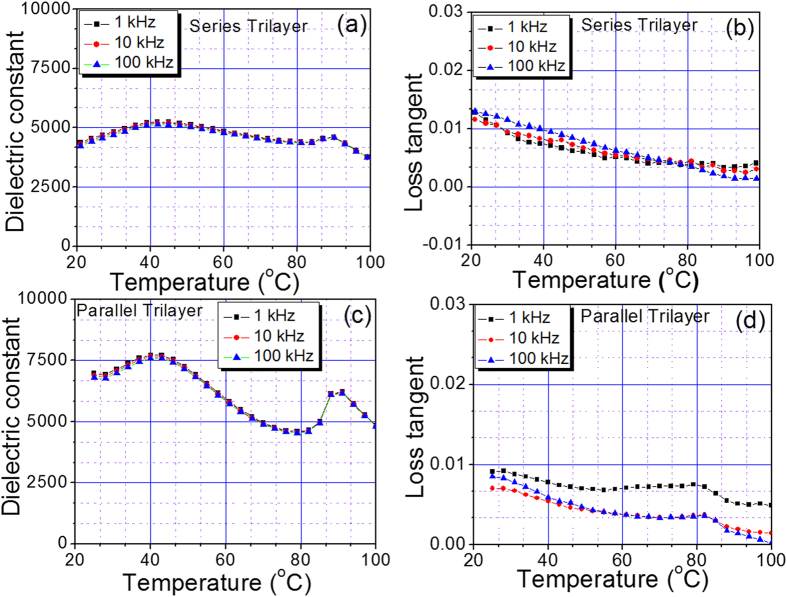
The phase transition behaviour for trilayer laminate composites. The dielectric constant and loss tangent versus temperature plots at various frequencies for trilayer composites in: (**a**,**b**) series configuration, (**c**,**d**) parallel configuration.

Figure 3 shows the temperature dependent dielectric constant and loss tangent at various frequencies for bilayer composites in parallel and series configurations. In series configuration, the Curie point corresponding to constituent compositions was not prominent especially for heavily doped composition. However, on measuring the dielectric response of the composites in parallel configurations (Fig. 1a), the Curie peaks corresponding to both the compositions were quite prominent exhibiting high dielectric permittivity (ε_m_). The values of loss tangent (<0.8% for parallel and <0.15% for series configurations) were also quite low in the given temperature range. However, to reduce the temperature dependent dielectric response, we synthesized new trilayer composite having additional composition with different Curie temperature. In this configuration, BTBCN with higher Curie temperature was sandwiched between two Sn doped ceramic compositions. Figure 4 shows the dielectric response of these trilayer composites as a function of temperature at various frequencies in series and parallel configurations. The Curie peaks related to the constituent compositions were not very clear in series configuration. However, in parallel configuration, one was able to observe Curie peak with relatively high ε_m_ for BTBCN. Furthermore, Curie peaks corresponding to other two Sn doped compositions appeared to be merged resulting in a broad peak near 40 °C. The values of dielectric loss tangents (<1.0% for parallel and <0.15% for series configurations) for both the configurations were very small in the temperature range of measurements similar to the bilayer composites.

**Figure 5:**
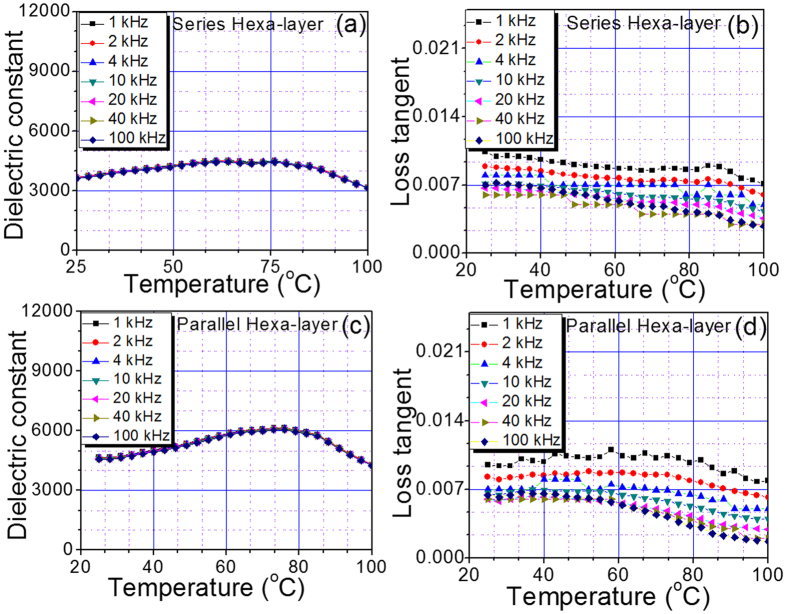
The temperature stability of the dielectric constant for hexa-layer laminate composites. The dielectric constant and loss tangent versus temperature plots at various frequencies for hexa-layer composites in: (**a**,**b**) series configuration, (**c**,**d**) parallel configuration.

In order to achieve improved temperature independent behavior, we synthesized multilayer ceramic composite with six constituent composition having strategically tuned Curie temperatures. Figure 5 shows the dielectric response as a function of temperature at various frequencies for a multilayer (hexa-layer) composite in series and parallel configurations. The dielectric constant as a function of temperature was found to depict a flat response without exhibiting clear peak due to merging of Curie temperature of constituent composition in series and parallel configurations. However, the average dielectric constant in parallel configuration was higher than that of its series counterpart. In both configurations, the dielectric loss tangent was quite small (<1%), which further demonstrated the practical relevance of these composites. Interestingly, these composites exhibited very small frequency dispersion of dielectric constant suggesting frequency independent behavior. This is quite interesting as in order to get a diffuse phase transition, researchers have synthesized relaxor type materials, but they are accompanied by extremely large frequency dependent behavior limiting their practical applications. Next, we present discussion about the observed dielectric response behavior for various multilayer structures and model the results using Landau theory of phase transformations that takes into consideration the electrostatic coupling between layers.

## Discussion

**Figure 6:**
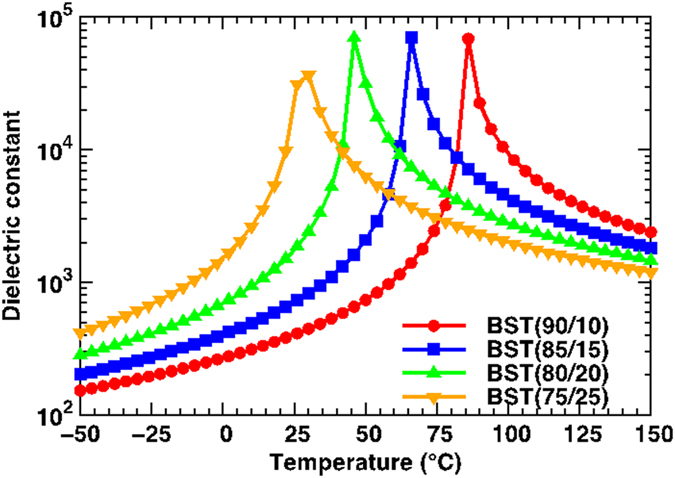
The phase transition behavior for various BST films having different compositions. The theoretical dielectric response as a function of temperature for monolithic, freestanding BST.

In order to describe the phase transition characteristics and the dielectric properties of the two different configurations of the bilayers and the compositionally graded multilayers as a function of temperature (*T*), we employed a nonlinear thermodynamic model taking into account the electrostatic interaction between layers^13^. We note that experimentally observed dielectric properties of ferroelectric multilayers have usually been described via a simple capacitor-in-series models^14^,^15^,^16^,^17^,^18^. This, however, is only an approximation and cannot explain several unique properties of ferroelectric multilayers^19^,^20^,^21^,^22^. Our approach was initially developed for freestanding and heteroepitaxial ferroelectric bilayers and has since been expanded to understand the electrostatic and electromechanical coupling in multilayer and polarization graded ferroelectrics^23^,^24^,^25^. The relevant thermodynamic, elastic, and electromechanical coefficients of Sn-modified 0.975BaTiO_3_-0.025Ba(Cu_1/3_Nb_2/3_)O_3_ (BTBCN) have not been determined experimentally. As such, to provide a meaningful explanation of experimental results, we considered Ba_*x*_Sr_1−*x*_TiO_3_ [BST *x*/(1 − *x*)] as a model system for the theoretical computations. The Curie temperature of BST (90/10) is around 90 °C, which is close to the *T*_C_ of BTBCN. An increase in the Sr concentration in BST results in a reduction of *T*_*C*_, which is similar to the Sn-modified systems^26^. The theoretical dielectric response of BST as a function of *T* obtained from the standard Landau theory of phase transformations is shown in Fig. 6 as a reference.

The total free energy density for a multilayer ferroelectric can be expressed as^27^:


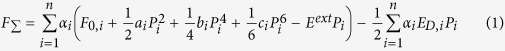


with


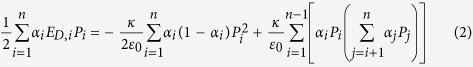


where *α*_*i*_ is the volume fraction of layer *i*, *F*_0,*i*_ is the free energy density at paraelectric phase, *P*_i_ is the polarization of layer *i* perpendicular to the electrode surface, *a*_*i*_, *b*_*i*_ and *c*_*i*_ are the dielectric stiffness coefficients of layer *i*. *E*^*ext*^ is the applied external electric field and *ED,i* is the depolarizing field in layer *i*.

For short-circuit boundary conditions, the potential drop between electrodes should be zero, or


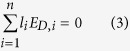


where *l*_*i*_ is the thickness of the *i*-th layer. The normal components of electric displacement field *D* on adjacent side of the boundary surface are related according to


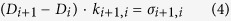


where *i* = 1,2, …, *n* − 1. Here, *k*_**i**+1,*i*_ is a unit vector normal to the surface, directed from media *i* + 1 to media *i* and *σ*_*i*+1,*i*_ is the macroscopic surface charge density at a given interface^28^. For *n*-layered case, we can rewrite Eqs. (4) as


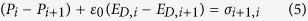


By introducing a scaling parameter *κ*, the surface charge density can be expressed as:


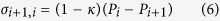


Therefore, 0 ≤ *κ* ≤ 1 entering Eq. 2 is a parameter that characterizes the strength of the coupling between layers and is related to charges that may exist in the multilayer composite. This can compensate for the depolarizing fields between layers that are generated due to the polarization mismatch. For *κ* = 0, individual layers are completely decoupled because the internal fields due to polarization variations are entirely compensated by free charges in the multilayer composite. For this case, the total free energy is simply the sum of the free energies of each layers that can be expressed as Landau expansions in the polarization (first term in Eq. 1) since Eq. 2 is equal to zero. For *κ* *=* 1, there are no (localized) compensating charges at the interlayer interfaces resulting in strong depolarizing fields as described by Eq. 2.

**Figure 7:**
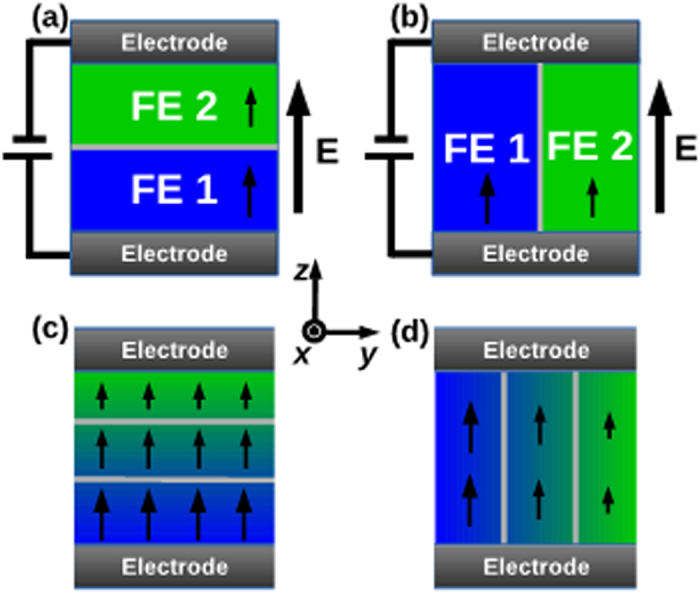
Schematic representations of the systems analyzed theoretically. (**a**) and (**b**) show two different configurations where the interface between the ferroelectric layers are parallel and perpendicular to the electrode, respectively. Configuration (**a**) corresponds to the condition where the polarizations in each layer are strongly coupled whereas in (**b**) the polarizations are weakly coupled. Likewise, (**c**,**d**) show compositionally graded constructs with the strongly and weakly coupled polarizations, respectively.

In our theoretical analysis, we considered two different configurations of ferroelectric bilayers and then expand this analysis to multilayered ferroelectrics with systematical variations in the composition. Figure 7a,b show schematic representation of the two bilayers that were analyzed. We show schematically that the interlayer interfaces between the ferroelectric layers are parallel and perpendicular to the electrodes in Fig. 7a,b, respectively. Figure 7a corresponds to the condition where the polarizations in each layer are strongly coupled whereas in Fig. 7b the coupling is weak if the easy axis of the polarization in the layers is parallel to the *z*-direction. As such, the bilayer configurations in Fig. 7a,b can be numerically modeled via the free energy functional given in Eq. 1 and 2 with *κ* ≈ 1 and *κ* ≈ 0 describing Fig. 7a,b, respectively. Eqs. 1 and 2 with *κ* ≈ 1 have been used to calculate dielectric properties of ferroelectric multilayers with an arrangement of layers as illustrated in Fig. 7a,c^29^,^30^. The justification for the approximation employed to describe the polarization and dielectric response of the configuration shown in Fig. 7b (and by extension, Fig. 7d) is provided by Chen *et al.*^31^ wherein the authors show that the “sideways” coupling of polarization in such a ferroelectric layer is insignificant if the layer thicknesses are much larger than the transition length of the polarization from one layer to another (of the order of 1 nm). This is indeed the case for the multilayers analyzed in this study. As such, the polarization in each layer for the geometry in Fig. 7b is very weakly coupled with *κ* ≈ 0 for which the total free energy can be expressed as the summation of the free energies of each layer. Figure 7c,d illustrate two different configurations of compositionally graded ferroelectric multilayer composites. Theoretically, these could be treated as extensions of the bilayer systems in Fig. 7a,b, respectively.

The small-signal average dielectric constant is calculated using


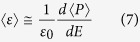


where


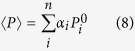


is the average polarization and 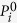 follow from the equations of state, *∂F*_Σ_/*∂P*_*i*_ = 0.

**Figure 8:**
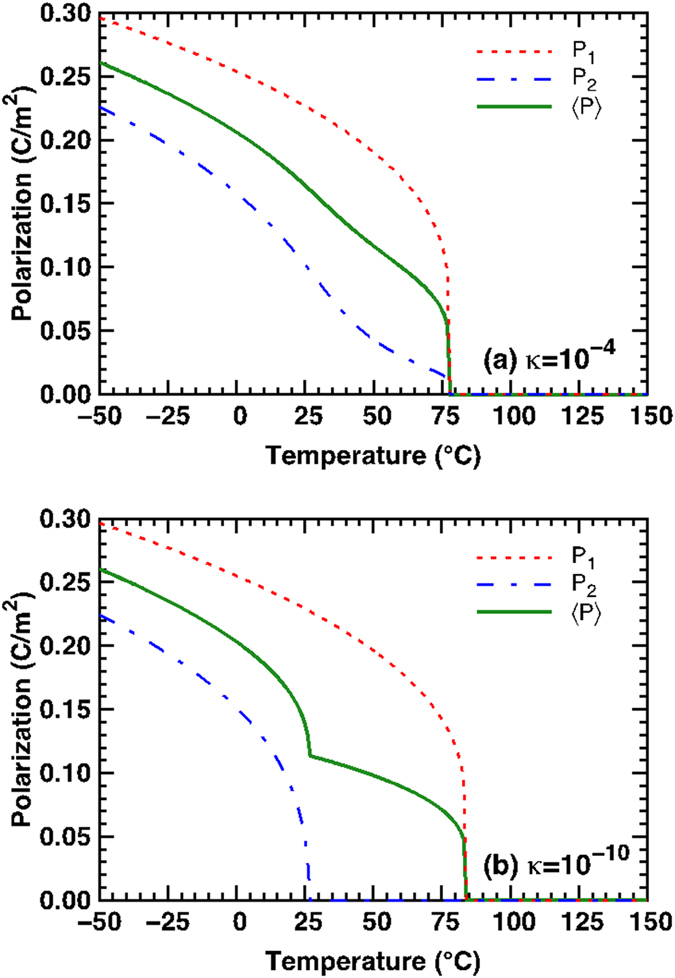
Effect of coupling on the polarization versus temperature plots for the BST bilayer system. BST (90/10) + BST (75/25) bilayer system with (**a**) strongly coupled polarization and (**b**) weakly coupled polarization, corresponding to configurations described in Fig. 2a,b respectively.

In Fig. 8a,b, we plot the equilibrium polarizations in the layers that make up the bilayers in Fig. 7a,b, respectively, as well as the average polarization of the bilayer. We choose an equi-fraction BST (90/10)/BST (75/25) bilayer in our analysis (bulk *T*_*C*_ = 82 °C and 26 °C, respectively). We note that properties of ferroelectrics, such as polarization, *T*_*C*_ and dielectric response, are extremely sensitive to the processing/deposition procedures and conditions. While extremely high-quality ceramic multilayers can be produced as shown in this study, it is difficult to compare the properties of interlayer interfaces in such samples to “perfect” heteroepitaxial, ultra-thin heterostructures^32^. Considering the mixed ionic and covalent interatomic bonding in perovskite ferroelectrics, their susceptibility to the formation of oxygen vacancies, and the presence of other structural defects such as impurities, dislocations, grain boundaries which have stress fields that generate commensurate polarization variations, the coupling between interlayers becomes extremely complex^33^. Taking into account that the interfaces in bulk ceramic samples are less than perfect (*κ* ≈ 1—no space charges), we take *κ* = 10^−4^ for configuration of Fig. 7a, which corresponds to a modest, space charge density (0.05 ~ 0.15 C/m^2^). Due to the coupling, equilibrium polarizations 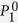 and 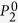 gradually decrease and vanish around the same temperature (approximately 78 °C). We note that Curie temperature of BST (90/10) layer does not vary significantly from monolayer (*T*_C_ ≈ 82 °C) to bilayer (*T*_C_ ≈ 78 °C) system but *T*_C_ of BST (75/25) shifts from 26 °C in a monolayer to 78 °C in bilayer case. In Fig. 8a, the average polarization smoothly decreases until the sudden drop at *T*_*C*_ of the bilayer. On the other hand, for the highly decoupled case *κ* = 10^−10^ ≈ 0 corresponding to Fig. 8b, 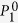 and 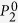 in bilayer system display similar phase transformation characteristics of bulk BST (90/10) and BST (75/25) with *T*_C_ ≈ 82 °C and 26 °C, respectively. The weakly coupled bilayer system behaves as the combination of two independent freestanding single layers as evident from the average polarization in Fig. 8b.

**Figure 9:**
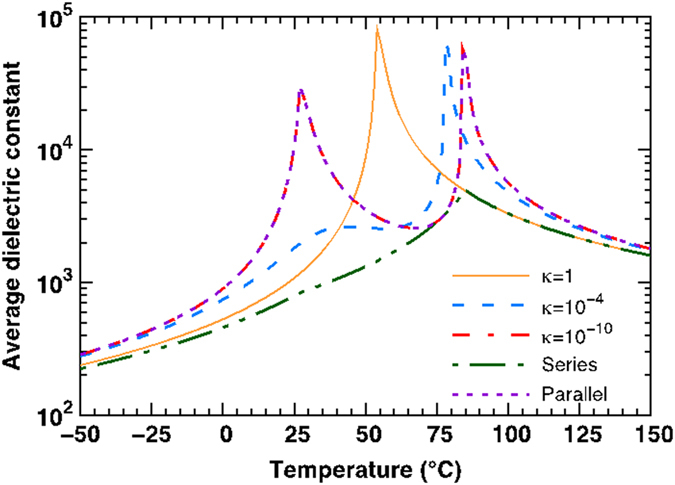
The magnitude of the coupling modulates the temperature dependence of the average dielectric response. The average dielectric constant of a freestanding, stress-free, equi-fraction Ba_0.9_Sr_0.1_TiO_3_/Ba_0.75_Sr_0.25_TiO_3_ bilayer is computed as a function of temperature. *κ* = 1, 10^−4^, and 10^−10^ represent the strongly coupled, weakly coupled, and decoupled behavior of bilayer system, respectively. The approximations using conventional formulas for capacitor-in-series (Series) and capacitor-in-parallel (Parallel) are shown as well.

The dielectric properties of the two bilayers in Fig. 7a,b follow from the polarization response shown in Fig. 8a,b. In Fig. 9, we computed the average dielectric response for the freestanding, stress-free, equi-fraction Ba_0.9_Sr_0.1_TiO_3_/Ba_0.75_Sr_0.25_TiO_3_ bilayer as a function of temperature to investigate the effect of the interlayer coupling. For 1 ≥ *κ* ≥ 10^−3^, the coupling strength is relatively strong. As such, the bilayer system behaves as if it were a uniform ferroelectric material with a single sharp maximum in the dielectric behavior *vs. T* corresponding to approximately the average composition of the bilayer^27^. For *κ* = 10^−4^, the interlayer interface is slightly decoupled and the average dielectric response is smeared out over a temperature range instead of a *λ*-type response for *κ* ≈ 1. For *κ* ≈ 0, there are two distinguishable peaks in the dielectric response corresponding to bulk BST (90/10) and BST (75/25). In other words, the layers in such a geometry (having *κ* ≈ 0) are entirely decoupled and standard relations for capacitors-in-parallel (Parallel) apply. The dielectric response using capacitors-in-series (Series) formula is also shown for comparison. The differences of the approximation using proposed nonlinear thermodynamics model and conventional capacitor formula are significant as expected^27^. Here, our theoretical model was successfully applied to understand dielectric response behavior of compositionally graded multilayer structures as shown in Fig. 7c,d. The trend of variations, of temperature dependent dielectric response of bilayer, with series and parallel configurations, were found to be similar to the theoretically predicted dielectric response behavior. As discussed previously, in series configurations, the bilayer specimen was found to exhibit only one prominent peak due presence of finite coupling at the interface. However, in case of the parallel configuration, Curie peaks due to both the constituents were prominent suggesting negligible coupling between the constituent layers. Similar behavior was observed for trilayer and multilayer composites in series and parallel configurations. Here, we experimentally demonstrated that by designing ceramic composite with strategically tuned phase transitions, high dielectric constant can be achieved over a wide temperature range. The presence of coupling, among constituent layers (depending on the magnitude of *κ*) of the composite, was found to modulate dielectric responses of the composites. Based on the theoretical results, the bilayer systems with parallel configurations were expected to be characterized by negligible value of *κ* (which defines the presence of the charges at the interface) and thereby small coupling between the layers. Moreover, the low loss in multilayer architectures could also be attributed to the interlayer interfaces that would trap mobile point defects to minimize the polarization difference and reduce the coupling. We believe this coupling will be significantly stronger in multilayers of monodomain and fully poled single crystals.

## Conclusions

We successfully fabricated highly dense multilayer ceramic composite structure with crack-free interface. These composites were fabricated using novel dielectric compositions having strategically tuned Curie temperatures. The dielectric response of these composites was different in series and in parallel configurations. The multilayer specimen, with series configurations, demonstrated enhanced dielectric response over a wide temperature range. We further modelled the results to provide fundamental understanding of this behavior. Theoretical calculations indicated that the finite coupling at the interface modulates the dielectric responses in series and in parallel configurations. These results further suggest that the high value of dielectric constant over a wide temperature range can be obtained through precise control of the interfacial coupling. The present work is expected to be helpful in designing advanced capacitive components for the future generations of the electronic devices.

## Methods

### Sample preparation

The 0.975BaTiO_3_–0.025Ba(Cu_1/3_Nb_2/3_)O_3_ (BTBCN) ceramics were synthesized using conventional mixed oxide processing method. Stoichiometric concentrations of CuO (Alfa Aesar, 99.0%), TiO_2_ (Alfa Aesar, 99.5%), Nb_2_O_5_ (Alfa Aesar, 99.5%) and BaCO_3_ (Alfa Aesar, 99.8%) were mixed by ball milling for 24 h with ZrO_2_ balls in polyethylene bottle and calcined in the range of 800–1000 °C for 2 h. Calcined powder was again ball milled to achieve homogeneous powder. In order to synthesize Sn-doped 0.975BaTi_1−y_Sn_y_O_3_-0.025Ba(Cu_1/3_Nb_2/3_)O_3_ ceramics (y = 0.015, 0.025, 0.035, 0.05, 0.06); stoichiometric concentration of SnO_2_ (Alfa Aesar, >99%) was added to the base matrix before first ball milling. These ceramics powders were mixed with the binder system and ball-milled for 24 h to get homogenous slurry. This slurry was tape casted using doctor blade with the blade height of 400 μm resulting in 50 μm thickness of the dried green tape. These tapes were cut in desired shape and laminated together with different compositions to achieve the desired thickness. After binder burnout, sintering was performed at 1350 °C for 2 h. The total thickness of various multilayer compositions was kept around 2.0 mm irrespective of the number of layers (e.g. bilayer had two layers with each layer having thickness of 1.0 mm). To achieve 2.0 mm thickness of sintered capacitor, almost 70 tapes were stacked together. The variations in the thickness of layers for the different compositions were achieved by controlling the number of green tapes in the stack. The geometry of the parallel and series capacitors were kept similar with the dimension of the 3 × 3 × 2 mm^2^ and therefore similar aspect ratio.

### Microstructure and Morphology

The interface and morphology of the sintered samples was observed using a LEO Zeiss 1550 (Zeiss, Munich, Germany) scanning electron microscope, as shown in Figure-S1 (Supplementary information) for a trilayer specimen. One can clearly see gradient in the microstructure across the cross-section of the specimen with crack-free interface (Fig. S1). The small elemental compositional difference between constituent layers in different composites was a primary reason for achieving crack-free interface. Moreover, the smaller compositional difference between the layers made it difficult to detect compositional variation using elemental dispersive spectroscopy (EDS).

### Dielectric measurements

For electrical measurements, silver paste (DuPont 7713) was applied on the surfaces (parallel and series) of sintered and polished ceramic samples and fired at 650 °C. Dielectric constant and tangent loss factor were measured as a function of temperature at selected frequencies using an inductance-capacitance-resistance (LCR) meter (HP 4284A), connected to a computer controlled high temperature furnace.

## Additional Information

**How to cite this article**: Maurya, D. *et al.* A new method for achieving enhanced dielectric response over a wide temperature range. *Sci. Rep.* **5**, 15144; doi: 10.1038/srep15144 (2015).

## Supplementary Material

Supplementary Information
