# Supplementary material for: A new method for achieving enhanced dielectric response over a wide temperature range
Source: Sci Rep. 2015 Oct 19;5:15144. doi: 10.1038/srep15144 (PMC4609923; doi:10.1038/srep15144)
Supplement: Supplementary Information [file srep15144-s1.pdf]

A new method for achieving enhanced dielectric response over a wide temperature range : Supplementary information

Deepam Maurya[1], Fu-Chang Sun[2], S. Pamir Alpay[2], and Shashank Priya[1*]*

[1]*Bio-inspired Materials and Devices Laboratory (BMDL), Center for Energy Harvesting Materials and Systems (CEHMS), Virginia Tech, 24061 USA*

[2] *Department of Materials Science & Engineering, Department of Physics, Institute of Materials Science, University of Connecticut, Storrs, CT 06269-3136, USA*

The scanning electron microscopy (SEM) was used to investigate the morphology of the composite specimens. All the types of the composite specimens considered in this work were found to show high relative density and crack-free interfaces. Here, we provide a representative SEM micrographs of a trilayer composite specimen. Fig. S1 shows the morphology of a trilayer composite across the interface. One can clearly observe two crack-free interfaces, suggesting high quality ceramics composites. Achieving crack-free interface was quite interesting considering a large gradient in the grain size of various layers having different compositions.

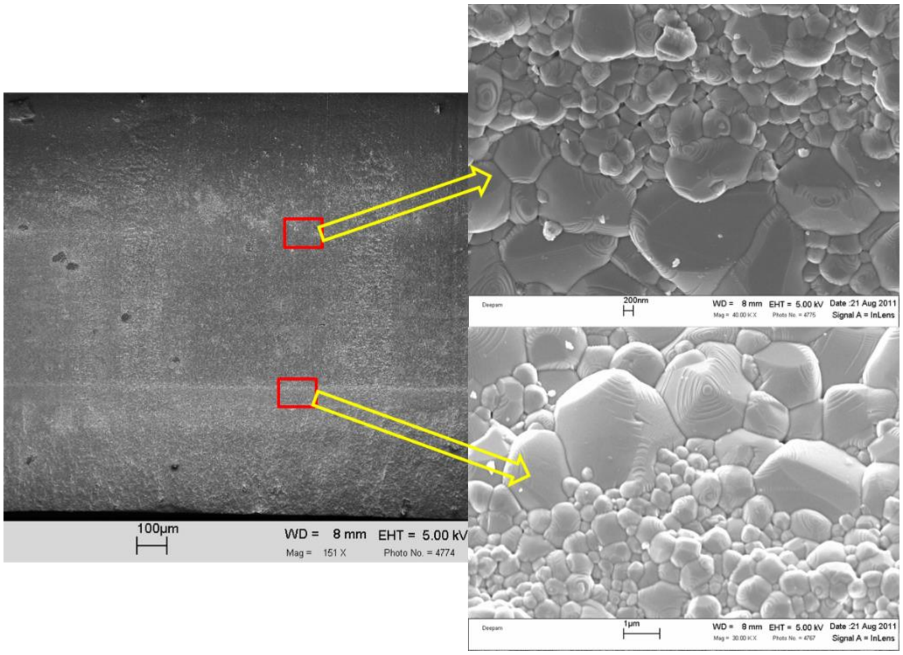

Figure-S1: SEM micrographs of a trilayer composite interface. Please note that all the composite samples with different schemes were found to have similar crack free interfaces.
